# miRSystem: An Integrated System for Characterizing Enriched Functions and Pathways of MicroRNA Targets

**DOI:** 10.1371/journal.pone.0042390

**Published:** 2012-08-01

**Authors:** Tzu-Pin Lu, Chien-Yueh Lee, Mong-Hsun Tsai, Yu-Chiao Chiu, Chuhsing Kate Hsiao, Liang-Chuan Lai, Eric Y. Chuang

**Affiliations:** 1 YongLin Biomedical Engineering Center, National Taiwan University, Taipei, Taiwan; 2 Bioinformatics and Biostatistics Core, Center of Genomic Medicine, National Taiwan University, Taipei, Taiwan; 3 Graduate Institute of Biomedical Electronics and Bioinformatics and Department of Electrical Engineering, National Taiwan University, Taipei, Taiwan; 4 Institute of Biotechnology, National Taiwan University, Taipei, Taiwan; 5 Department of Public Health, National Taiwan University, Taipei, Taiwan; 6 Graduate Institute of Physiology, National Taiwan University, Taipei, Taiwan; The Roslin Institute, University of Edinburgh, United Kingdom

## Abstract

**Background:**

Many prediction tools for microRNA (miRNA) targets have been developed, but inconsistent predictions were observed across multiple algorithms, which can make further analysis difficult. Moreover, the nomenclature of human miRNAs changes rapidly. To address these issues, we developed a web-based system, miRSystem, for converting queried miRNAs to the latest annotation and predicting the function of miRNA by integrating miRNA target gene prediction and function/pathway analyses.

**Results:**

First, queried miRNA IDs were converted to the latest annotated version to prevent potential conflicts resulting from multiple aliases. Next, by combining seven algorithms and two validated databases, potential gene targets of miRNAs and their functions were predicted based on the consistency across independent algorithms and observed/expected ratios. Lastly, five pathway databases were included to characterize the enriched pathways of target genes through bootstrap approaches. Based on the enriched pathways of target genes, the functions of queried miRNAs could be predicted.

**Conclusions:**

MiRSystem is a user-friendly tool for predicting the target genes and their associated pathways for many miRNAs simultaneously. The web server and the documentation are freely available at http://mirsystem.cgm.ntu.edu.tw/.

## Introduction

MicroRNAs (miRNAs) are short, non-coding RNAs which regulate their corresponding target genes through post-transcriptional repression [1]. It has been shown that miRNAs play important roles in many cellular processes such as stress responses [2], hematopoiesis [3], radiation responses [4], and the immune system [5]. A growing body of evidence has indicated that dysregulation of miRNAs results in several diseases including cardiovascular disease [6], type 2 diabetes [7], and multiple cancers [8]. Therefore, treatment of those diseases may be improved with better understanding of how miRNAs participate in regulation of gene expression during pathogenic processes.

With the advancement in microarray and next generation sequencing technologies, researchers are able to investigate miRNA expression profiles *en masse* at a lower cost. To evaluate the associations between mRNA and miRNA, several useful prediction tools for miRNA target genes have been developed. For example, miRanda and TargetScan both predict miRNA target genes by seed-matching and three prime untranslated region (3′UTR) pairing [9], [10], [11], [12], and DIANA-microT develops a dynamic programming algorithm to calculate scores based on affinity of the interactions between miRNAs and gene targets [13], [14]. MirBridge explores regulatory miRNAs by considering whether their functional binding sites were enriched among a gene set with pre-defined biological functions [15], whereas PicTar claims miRNA-gene interaction pairs according to the binding probability between mature miRNAs and the 3′UTR of the gene target [16]. Moreover, rna22 identifies putative miRNAs by mapping their binding sites through a pattern-based approach [17], and PITA incorporates free energy intake and cost to evaluate the interactions between miRNAs and gene targets [18]. Challenges arise, however, when predictions are inconsistent across multiple algorithms. Discrepancies may be mainly attributed to the use of different modeling formulas, which consider distinct physical and biochemical characteristics.

Two elementary methods to summarize the prediction results from different algorithms are union and intersection analyses, but the performance of such approaches is usually not very good. For a specific miRNA or a set of co-expressed miRNAs, combining prediction results across different algorithms into a union set often leads to numerous target genes with high false-positive rates. On the other hand, selecting predicted targets in common from the intersection set usually results in very few genes due to stringent selection criteria. To overcome these challenges, one possible strategy is to integrate the results by using a voting scheme and establishing a statistical threshold based on existing data to identify the optimal cutting point. After identification of potential target genes, it is well known that pathway analyses can help to reveal the biological functions regulated by given miRNAs [19], [20]. Yet, one major limitation in these algorithms is that the raw expression values of each gene are only used to identify the ranking or statistical differences among them [21]. Subsequent over-representation approaches through identified genes do not incorporate the raw expression values to pinpoint enriched functional pathways. However, since the effects of different miRNAs on gene expression are not equivalent, considering miRNA expression levels during pathway analyses may further improve the robustness of the results. Performing these pathway analysis methods on identified miRNA target genes facilitates characterization of the biological roles of queried miRNAs and addresses how they participate in transcriptional regulation.

In addition to discrepancies in the prediction of target genes, the integration of different algorithms poses another major challenge: the nomenclature of miRNAs in commonly used databases (such as miRBase [www.mirbase.org]) has changed over time. Not only have some miRNAs been retired due to irreproducibility of the original results, but a single miRNA may also have multiple names in different versions of the database (Table S1 and Figure S1). Similar to the chaos caused by multiple names for the same gene, such inconsistent annotation is error-prone and makes it difficult for users to perform a correct search and analysis. Moreover, with the ongoing study of miRNAs, many novel miRNAs are included in newer releases of miRBase. Therefore, a systematic mapping of miRNA sequences to the latest nomenclature is a prerequisite to do any analysis.

In this article, we present miRSystem, a web-based system that matches queried miRNAs with the latest annotation and identifies the biological functions/pathways regulated by miRNAs based on the enriched functions of their target genes. Searching for the enriched pathways of miRNAs can be implemented by bootstrap approaches or incorporating miRNA expression values. The results of enrichment analysis and gene-gene interaction maps are shown in tabular or graphical output.

## Methods

### Overview of miRSystem workflow

**Figure 1:**
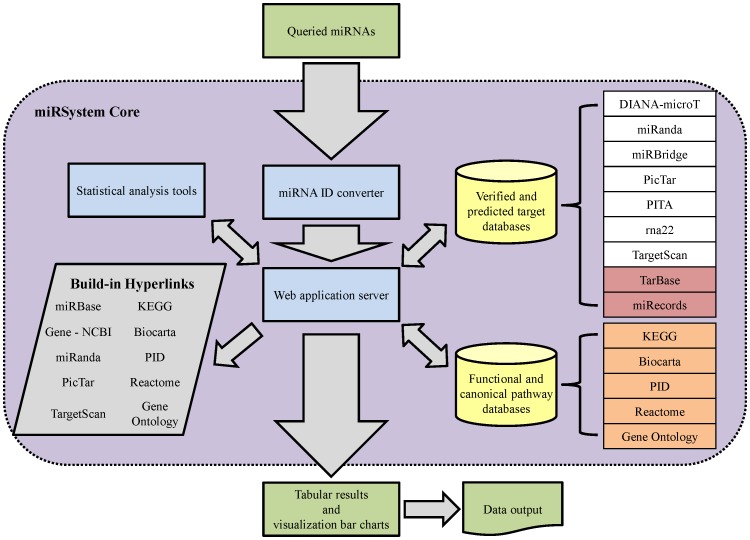
An overview of miRSystem. The green boxes indicate the data input and output. The purple rectangle with a dotted outline represents the miRSystem core. The three major components of miRSystem (blue boxes) are the miRNA ID converter, statistical analysis tools, and web application server. The target prediction algorithms (upper yellow cylinder) constitute 7 programs (white boxes) and 2 experimentally validated data sets (red boxes). The pathway prediction module (lower yellow cylinder) includes the information from 5 databases (orange boxes).

An overview of the miRSystem database and modules is illustrated in Figure 1. Briefly, queried miRNAs are first converted to the latest miRBase annotation (currently version 17). Subsequently, to identify significantly enriched signaling pathways, several statistical approaches are provided, including O/E ratios of gene targets, hypergeometric *P*-value and empirical *P*-value from permutation. All analyses are executed by the miRSystem application server through a graphical user interface. Results are displayed in tabular or graphical format, and hyperlinks to the original data sources are also provided.

### Database construction and contents

Currently, miRSystem can analyze two species: *Homo sapiens* and *Mus musculus*. Seven algorithms predicting miRNA targets, DIANA-microT [13], [14], miRanda [9], [22], [23], mirBridge [15], PicTar [16], PITA [18] , rna22 [17] , and TargetScan [10], [11], [12], and two experimentally validated databases, TarBase [24] and miRecords [25], of miRNA target genes were included. The details of each algorithm and database are summarized in Table S2. Five pathway databases, including Gene Ontology [26], KEGG [27], BioCarta, Pathway Interaction Database [28], and Reactome [29], were used to annotate the biological functions and canonical pathways of target genes.

A major challenge in establishing the miRSystem database is the inconsistent miRNA IDs used across different algorithms and data sources (Table S1). For example, hsa-miR-24-1* in version 17 of miRBase has another official name, hsa-miR-189, before version 9.2. Such multiple names for the same miRNA can cause errors and produce misleading results. To overcome this problem, we developed miRConverter, which collects all versions of annotation in miRBase, converts queried miRNAs into the latest version, and removes retired miRNA. Lastly, gene names from different algorithms and pathways were also unified to the standard HUGO gene symbols.

### Database characteristics in predicting miRNA-gene relationships

To identify the optimal threshold of multiple prediction algorithms, characteristics of predicted miRNA-gene relationships were summarized (Table S3). The “hit” represents the number of algorithms predicting the same miRNA-gene interaction pair. As shown in Table S3, almost 75% of miRNA-gene associations were predicted only by one algorithm, indicating that multiple algorithms must be combined in order to identify possible miRNA-gene associations. To balance the reliability of the predictions with a manageable number of records, the default parameter in miRSystem was set at three algorithm hits. Approximate 9% of the total predictions are analyzed when this setting is selected.

### Simulation of the null baselines for identifying biological functions that target genes are enriched

After identifying potential target genes, a hypergeometric test is used to characterize the functions of the target genes. Since raw *P*-values are easily affected by the number of genes of a given function/pathway, we also calculated an empirical *P*-value. The empirical *P*-values of each function/pathway were determined by ranking the enriched hypergeometric probability as compared with null baseline probabilities. The null baseline probability was established by randomly selecting a group of miRNAs, whose size ranges from 1 to 100, and using the default values in miRSystem to calculate the raw *P*-value for each function/pathway. The default analysis parameters in miRSystem were following: (a) O/E ratio = 2, (b) hit frequency = 3, and (3) validated miRNA-gene pairs were included for analyses. This simulation process was repeated 1,000 times to build the null baseline.

### Weighted pathway-ranking method for identifying enriched biological functions

If miRNA expression values were available, the ratios of experimental group to control group for queried miRNAs were incorporated into an additional weighted pathway-ranking method to identify the enriched biological functions. First, for a given set of miRNAs, expression levels of the miRNAs were required to be used as the weight. The weight for one miRNA was calculated by dividing its absolute expression value by the absolute sum of the expression values of all input miRNAs. Next, after identifying the target genes by the selected prediction algorithms, for each functional category, the ranking score was obtained by summation of the weight of its miRNA times its enrichment −log (P-value) from the predicted target genes 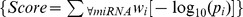. When equal weights are assumed, i.e., no differences in miRNA expression were considered, this weighted scoring method reverted to the previously described hypergeometric test procedure. In general, the results of these two algorithms are comparable; users can choose this method when expression values of their miRNAs are available.

## Results

### Website interface

**Figure 2:**
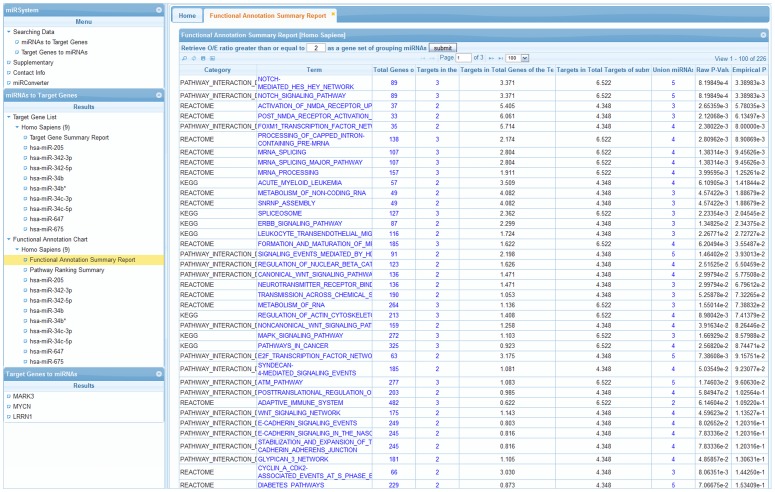
Graphical user interface in miRSystem. A hierarchical menu is shown in the left panel and the queried and analyzed results are shown in the main window.

miRSystem is a web-based system, which is implemented mainly by PHP5 language, a mySQL database management system, and a jQuery grid plugin. As shown in Figure 2, a hierarchical menu in the left panel lists the items of queried and analyzed data. After clicking, the results of each item are displayed in the main window in tabular format. Users can sort selected results in ascending or descending order, and all data can be exported in csv-format files. In general, the computation time of each function in miRSystem is on the order of minutes, depending on the complexity of the miRNA targets. The major functions provided in miRSystem are demonstrated in the following examples.

### Example 1: Conversion of miRNA IDs across different annotation versions

**Figure 3:**
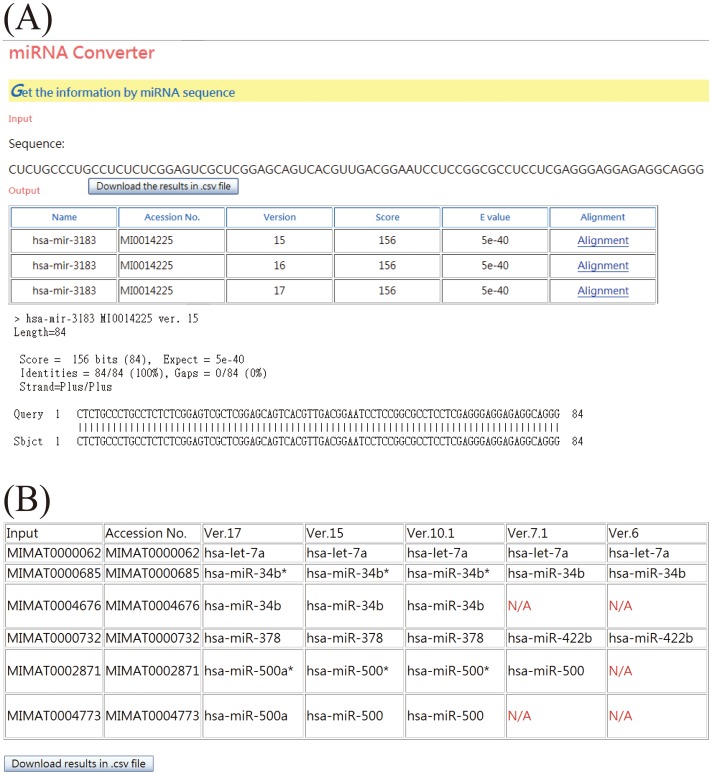
Screenshots of miRConverter. miRConverter allows two types of query format: (A) miRNA sequence and (B) accession number.

To reduce the potential discrepancies caused by multiple naming conventions used among different algorithms and databases (Table S2), queried IDs are converted into the latest annotation in miRBase (currently version 17). Users can submit an miRNA ID or a sequence of mature miRNA (Figure 3A). The basic local alignment search tool (BLAST) is utilized to identify the miRNA IDs with the highest similarity as compared to the input miRNA sequences. Results with different versions of annotation are shown in a table (Figure 3B), which can be downloaded as a csv-format file.

### Example 2: Prediction of functions that grouped miRNAs are enriched

**Figure 4:**
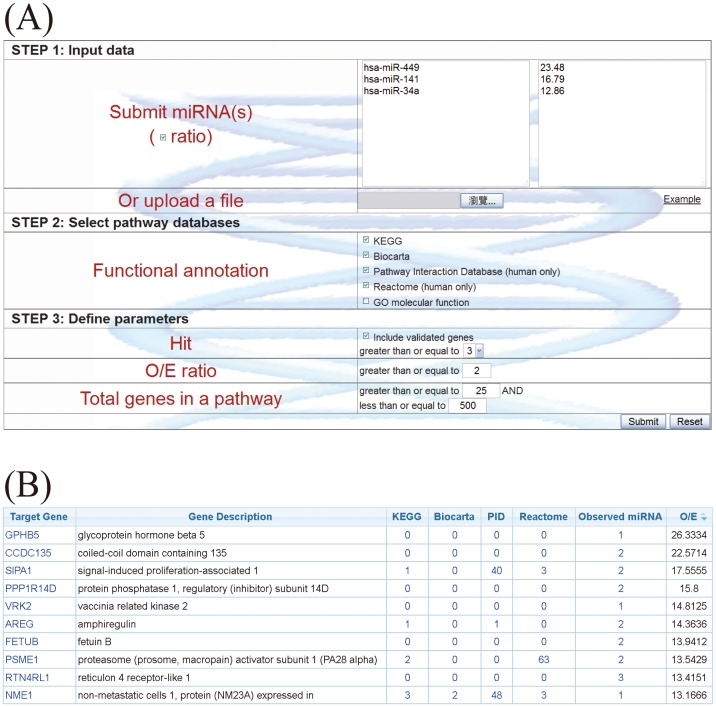
Identification of miRNA target genes in miRSystem. (A) The miRNA input interface. (B) Tabulated results of enriched pathways of miRNA target genes based on the consistency across multiple algorithms and observed/expected ratios.

**Figure 5:**
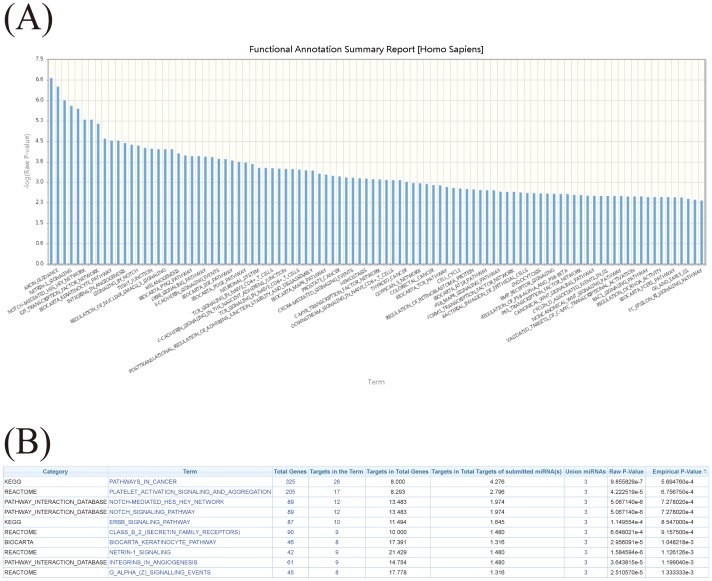
Results of pathway enrichment analysis. (A) The graphical output of enriched pathways of miRNA target genes. Y-axis: −log (*P*-value). (B) Tabulated results of enriched pathways of miRNA target genes using a pathway-ranking algorithm that incorporates weighting based on the expression levels of a given miRNA set.

**Figure 6:**
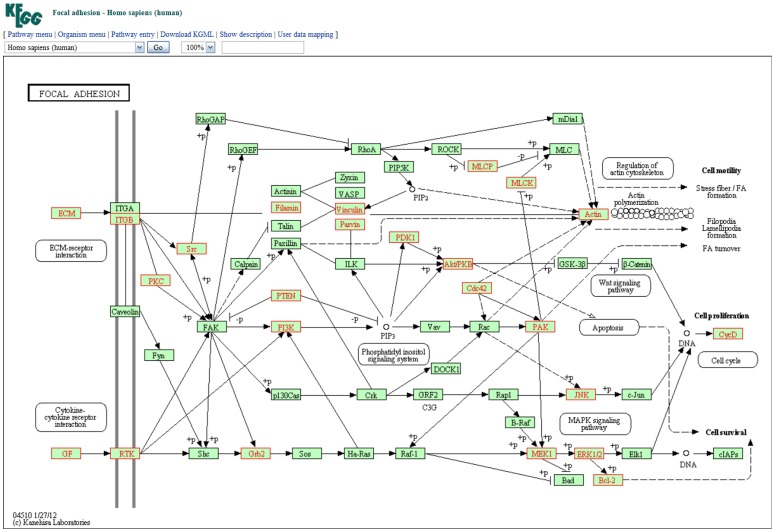
Illustration of embedded hyperlink to KEGG database. The genes highlighted in red color are predicted to be regulated by queried miRNAs.

**Figure 7:**
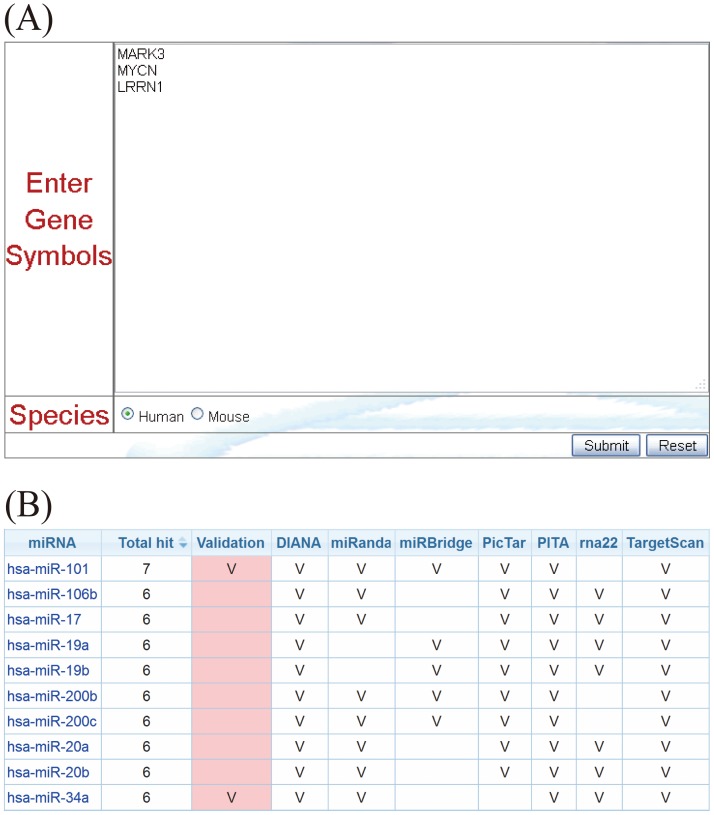
Identification of regulatory miRNAs from predicted target genes. (A) Gene input interface for query of regulatory miRNAs. (B) Tabulated results of prediction of regulatory miRNAs.

Once users provide a list of miRNAs and, optionally, their expression ratios (Figure 4A), as illustrated in example 1, these miRNAs are first converted to the latest annotation in miRBase. Users can choose different parameters to identify possible miRNA target genes by considering multiple prediction algorithms simultaneously (Figure 4A). After the miRNA target genes are identified, to identify the pathways in which the target genes participate, an observed to expected (O/E) ratio is first calculated. The observed identification probability for a given gene is the proportion of the queried miRNA(s) which are predicted to target that gene, whereas the expected probability is the proportion of all miRNAs in the miRSystem database predicted to target that gene, i.e., the number of target gene-miRNA pairs deposited in the miRSystem database. This expected probability represents the chance of one gene being randomly selected by miRNAs. The default O/E ratio is 2, but users are able to adjust this value (Figure 4B). Next, cumulative hypergeometric distribution is used to characterize enriched biological functions/pathways of these predicted target genes. Graphical results are sorted by −log(*P*-value) to facilitate identification of functional pathways that these input miRNAs are involved (Figure 5A). Additionally, empirical *P*-values indicate the probability of identifying pathways by random chance (Figure 5B), and hyperlinks to original pathway databases are also embedded (Figure 6). Combining the pathway enrichment results from raw *P*-values and empirical *P*-values may help to reduce the false positive and reveal the biological functions indeed regulated by queried miRNAs. Moreover, users are able to explore possible regulatory miRNAs by inputting user's favorite genes (Figure 7A). The predicted miRNAs by which algorithms are also indicated in a summary table (Figure 7B).

### Example 3: Demonstration of analysing datasets in public domain

To demonstrate the usefulness of miRSystem, we analyzed two microarray datasets with both mRNA and miRNA data (GSE16558 and GSE19536) in the Gene Expression Omnibus (GEO) [30], [31], [32]. For GSE16558, there were 60 multiple myeloma samples and 5 normal controls. Three miRNAs were significantly (*P*-value<5.1*10^−4^) up-regulated in multiple myeloma using unpaired t-tests with unequal variance (Table S4A). Afterwards, 1,527 genes were indicated as the potential target genes of these 3 miRNAs using the default values in miRSystem. Since miRNAs were expected to down-regulated their target genes, proportion of down-regulated genes predicted by miRSystem (48.53%) was higher than that in the whole genome (38.66%; Table 1 and Figure S2A).

**Table 1:** Significant miRNAs and identified target genes of GSE16558 and GSE19536^a^.

	GSE16558		GSE19536	
	Whole Genome	3 miRNAs	Whole Genome	5 miRNAs
	20,177 genes	1,527 genes	30,981 genes	1,425 genes
Number of down-regulated genes				
Down-regulation <1.0 fold	7801 (38.66%)	741 (48.53%)	16064 (51.85%)	789 (55.37%)
Down-regulation <0.67 fold	2183 (10.82%)	226 (14.80%)	782 (2.52%)	46 (3.23%)
Down-regulation <0.5 fold	766 (3.80%)	76 (4.98%)	203 (0.66%)	16 (1.12%)

a The proportion of down-regulated genes was calculated relative to the total number of genes under each condition.

Similarly, for GSE19536, unpaired t-tests were done to identify miRNAs that were up-regulated in breast cancer patients with estrogen receptor–positive tumors. Six miRNAs were significantly (*P*-value<5 * 10^−9^) up-regulated (Table S4B). However, because hsa-miR-29c* was a newly identified miRNA, no gene target predictions for hsa-miR-29c* were recorded in miRSystem. Therefore, the following analyses only focused on the remaining 5 miRNAs. A total of 1,425 genes were identified as the potential targets of the 5 miRNAs using the default values in miRSystem. As shown in Fig. S2B, the proportion of target genes that was down-regulated (55.37%) was slightly higher than that in the whole genome (51.85%; Table 1 and Figure S2B). These results suggest that miRSystem was able to identify the possible target genes of a group of miRNAs by consensus among multiple prediction algorithms.

After identifying the potential target genes, we further explored the pathways in which these genes participate (Tables S5 and S6). In GSE16558, the target genes of the 3 identified miRNAs were involved in cancer, platelet activation signaling and aggregation, and the notch signaling pathway (Table S5A). These pathways were expected in patients with multiple myeloma. Dysregulation of the notch signaling pathway has been demonstrated as an important step in transforming normal cells into tumors [33], [34], [35]. Platelet activation and aggregation are highly associated with thrombosis [36], [37], which is a causative factor leading to multiple myeloma [38], [39], and the changes in several cancer-related genes curated by KEGG were possibly regulated by these miRNAs.

Similarly, in GSE19536, several previous studies have reported that breast cancer cells undergo distinct apoptosis procedures based on estrogen receptor status [40], [41]. The genes involved in the apoptosis pathway were thus expected to show differential expressions in breast cancer patients with estrogen receptor-positive tumors (Table S6). Regarding the axon guidance pathway, many molecules play important roles in driving tumorigenesis and progression [42], [43], [44].

The enriched pathways identified by considering miRNA expression levels were generally similar to that obtained from hypergeometric tests in GSE19536 data set (Table S6), but differences were observed in GSE16558 (Table S5). This may be attributed to the fact that the fold changes in miRNA expression were substantially larger in GSE16558 than in GSE19536 (Table S4). In conclusion, these results suggested that characterization of dysregulated cellular functions and pathways among multiple miRNAs using miRSystem was able to identify promising targets with meaningful biological implications.

## Discussion

With the rapid growth of high-throughput sequencing technologies and massive genetic data sets, many novel miRNAs have been identified, and many computational algorithms have been developed to predict possible miRNA target genes by considering distinct physical, chemical, and biological characteristics. However, as shown in Table S3, inconsistent miRNA target gene prediction results across different algorithms pose a major challenge. Taking hsa-miR-590-3p for an example, if we used the union method to summarize the gene prediction results, a total of 7,421 genes would be indicated as potential targets, which is a dramatically large number to be validated experimentally. Under the default settings in miRSystem, only 1,383 genes were suggested as possible target genes of hsa-miR-590-3p. Therefore, miRSystem simultaneously considers multiple prediction algorithms to reduce the false positive rate.

Regarding the annotated miRNA databases and the prediction algorithms deposited in miRSystem, several features are worth mentioning. We aimed to provide the original prediction scores or probabilities of the miRNA-gene interaction pairs from the respective prediction algorithms, since this information is helpful to users in selecting reliable prediction targets. Yet, such data were not available for all the prediction algorithms, and thus in those cases we could only embed hyperlinks to their original websites. In addition, these prediction algorithms were developed based on distinct statistical models and evaluation systems respectively, which made it a challenge to identify a unify function in integrating the prediction results. Currently, a voting scheme was used in miRSystem to identify possible miRNA-gene interaction pairs, but this voting scheme may be improved by a multiple scoring system using fusion analysis method or rank-score characteristics function [45], [46], [47]. To further enlarge the miRSystem database, we will continue to collect and incorporate accurate miRNA target gene prediction algorithms. In addition to *Homo sapiens* and *Mus musculus*, the sequences of miRNAs and their target genes have been rapidly accumulated in other species, such as rat, fly and many plants [22], [48], [49]. We propose to include these data in the future to further improve the usefulness of miRSystem and facilitate characterizing miRNA variations in those species.

## Conclusions

In this study, we present miRSystem, a user-friendly, web-based system designed to perform miRNA target gene analysis and prediction of biological functions and canonical pathways of miRNAs and their target genes. Several prediction algorithms and experimentally validated data sources were integrated into the miRSystem to reduce false positive predictions. A miRNA ID converter among different miRBase versions was utilized to remove the potential discrepancies in nomenclature. Two enrichment algorithms—with or without consideration of miRNA expression changes—were incorporated to explore dysregulated biological functions/pathways. To our knowledge, miRSystem is the first analytical tool that identifies miRNA target genes based on multiple algorithms simultaneously. We believe that the development of miRSystem will increase the accuracy of target gene analysis and facilitate the dissection and interpretation of the biological functions affected by miRNAs.

## Supporting Information

Figure S1 **Example of changes of miRNA names in different miRBase versions.** Hsa-miR-34b is MIMAT0000685 from version 6 to version 10, but is MIMAT0004676 after version 10.
(PDF)

Figure S2 **Heat map of identified target genes using MiRSystem.** The input data of each gene was normalized relative to its biological control samples. That is, for each gene, normal control or estrogen receptor-negative samples were used as a normalization baseline, so the mean probe intensity of them was subtracted from probe intensities in the multiple myeloma or estrogen receptor-positive samples. One-way hierarchical clustering with average linkage distance was performed on these transformed values in both datasets. (A) GSE16558, 1,527 genes. (B) GSE19536, 1,425 genes.
(PDF)

Table S1 **Conflicted miRNA IDs in different versions of miRBase**
(PDF)

Table S2 **Prediction algorithms and databases available in miRSystem**
(PDF)

Table S3 **The potential number of miRNA-gene pairs obtained with different combination of multiple algorithms**
(PDF)

Table S4 **Significantly expressed miRNAs**
(PDF)

Table S5 **Top 3 enriched pathways of the 3 miRNAs identified in GSE16558 by (A) functional annotation summary and (B) pathway ranking summary in miRSystem**
(PDF)

Table S6 **Top 3 enriched pathways of the 5 miRNAs identified in GSE19536 by (A) functional annotation summary and (B) pathway ranking summary in miRSystem.**
(PDF)
