# Supplementary material for: miRSystem: An Integrated System for Characterizing Enriched Functions and Pathways of MicroRNA Targets
Source: PLoS One. 2012 Aug 1;7(8):e42390. doi: 10.1371/journal.pone.0042390 (PMC3411648; doi:10.1371/journal.pone.0042390)
Supplement: Figure S1 — Example of changes of miRNA names in different miRBase versions. Hsa-miR-34b is MIMAT0000685 from version 6 to version 10, but is MIMAT0004676 after version 10. (PDF) [file pone.0042390.s001.pdf]

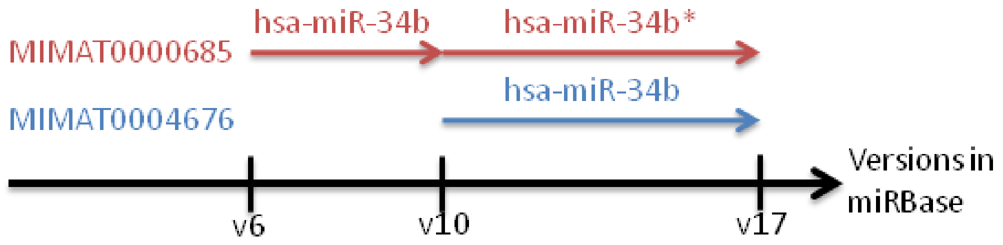

Figure S1 – Example of changes of miRNA names in different miRBase versions. Has-miR-34b is MIMAT0000685 from version 6 to version 10, but is MIMAT0004676 after version 10.
