# Supplementary material for: miRSystem: An Integrated System for Characterizing Enriched Functions and Pathways of MicroRNA Targets
Source: PLoS One. 2012 Aug 1;7(8):e42390. doi: 10.1371/journal.pone.0042390 (PMC3411648; doi:10.1371/journal.pone.0042390)
Supplement: Figure S2 — Heat map of identified target genes using MiRSystem. The input data of each gene was normalized relative to its biological control samples. That is, for each gene, normal control or estrogen receptor-negative samples were used as a normalization baseline, so the mean probe intensity of them was subtracted from probe intensities in the multiple myeloma or estrogen receptor-positive samples. One-way hierarchical clustering with average linkage distance was performed on these transformed values in both datasets. (A) GSE16558, 1,527 genes. (B) GSE19536, 1,425 genes. (PDF) [file pone.0042390.s002.pdf]

(A)

(B)

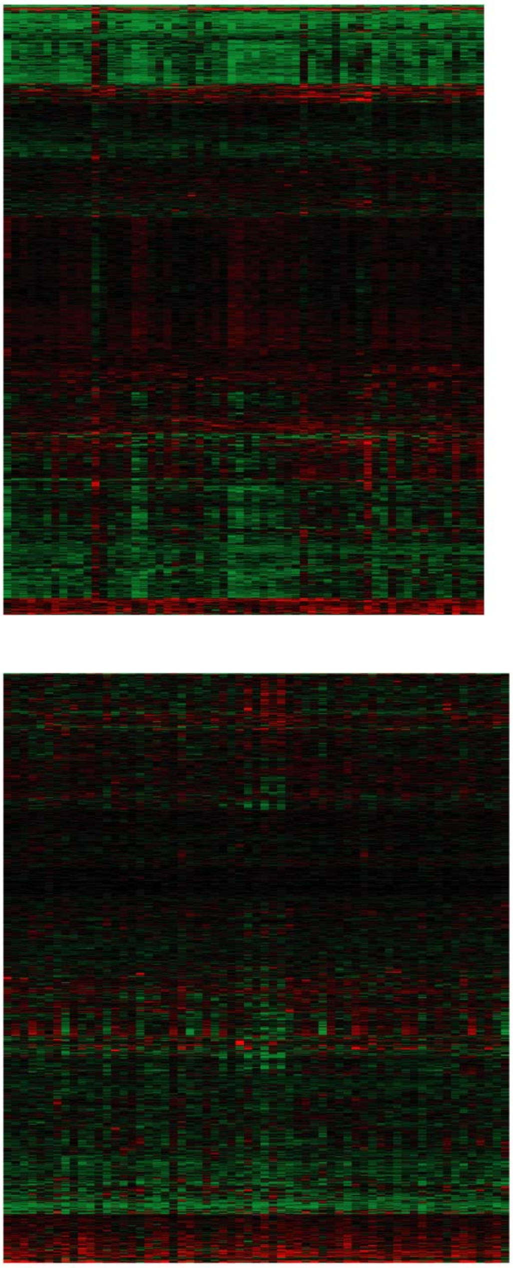

**Figure S2 –Heat map of identified target genes using MiRSystem.** The input data of each gene was normalized relative to its biological control samples. That is, for each gene, normal control or estrogen receptor-negative samples were used as a normalization baseline, so the mean probe intensity of them was subtracted from probe intensities in the multiple myeloma or estrogen receptor-positive samples. One-way hierarchical clustering with average linkage distance was performed on these transformed values in both datasets. (A) GSE16558, 1,527 genes. (B) GSE19536, 1,425 genes.
