# Supplementary material for: miRSystem: An Integrated System for Characterizing Enriched Functions and Pathways of MicroRNA Targets
Source: PLoS One. 2012 Aug 1;7(8):e42390. doi: 10.1371/journal.pone.0042390 (PMC3411648; doi:10.1371/journal.pone.0042390)
Supplement: Table S1 — Conflicted miRNA IDs in different versions of miRBase (PDF) [file pone.0042390.s003.pdf]

Table S1 – Conflicted miRNA IDs in different versions of miRBase

miRNA Accession	miRNA ID in version 9.2	miRNA ID in version 17	Sequence
MIMAT0000685	hsa-miR-34b	hsa-miR-34b*	UAGGCAGUGUCAUUAGCUGAUUG
MIMAT0004676	N/A	hsa-miR-34b	CAAUCACUAACUCCACUGCCAU
MIMAT0000750	hsa-miR-340	hsa-miR-340*	UCCGUCUCAGUUACUUUAUAGC[1]
MIMAT0004692	N/A	hsa-miR-340	UUAUAAAGCAAUGAGACUGAUU
MIMAT0000731	hsa-miR-378	hsa-miR-378*	CUCCUGACUCCAGGUCCUGUGU
MIMAT0000732	hsa-miR-422b	hsa-miR-378	ACUGGACUUGGAGUCAGAAGG[2]
MIMAT0001343	hsa-miR-425	hsa-miR-425*	AUCGGGAAUGUCGUGUCCGCCC
MIMAT0003393	hsa-miR-425-5p	hsa-miR-425	AAUGACACGAUCACUCCCGUUGA
MIMAT0002804	hsa-miR-488	hsa-miR-488*	CCCAGAUAAUGGCACUCUCAA
MIMAT0004763	N/A	hsa-miR-488	UUGAAAGGCUAUUUCUUGGUC
MIMAT0002813	hsa-miR-493 (in version 7.1)	hsa-miR-493*	UUGUACAUGGUAGGCUUUCAUU
MIMAT0003161	hsa-miR-493-3p	hsa-miR-493	UGAAGGUCUACUGUGUGCCAGG
MIMAT0002871	hsa-miR-500	hsa-miR-500a*	AUGCACCUGGGCAAGGAUUCUG
MIMAT0004773	N/A	hsa-miR-500 (in version 15)	UAAUCCUUGCUACCUGGGUGAGA
MIMAT0003257	hsa-miR-550	hsa-miR-550a*	UGUCUUACUCCCUCAGGCACAU
MIMAT0004800	N/A	hsa-miR-550 (in version 15)	AGUGCCUGAGGGAGUAAGAGCCC
MIMAT0003256	hsa-miR-589	hsa-miR-589*	UCAGAACAAAUGCCGGUUCCCAGA
MIMAT0004799	N/A	hsa-miR-589	UGAGAACCACGUCUGCUCUGAG
MIMAT0003261	hsa-miR-593	hsa-miR-593*	AGGCACCAGCCAGGCAUUGCUCAG C

MIMAT0004802	N/A	hsa-miR-593	UGUCUCUGCUGGGGUUUCU
MIMAT0003284	hsa-miR-616	hsa-miR-616*	ACUCAAAACCCUUCAGUGACUU
MIMAT0004805	N/A	hsa-miR-616	AGUCAUUGGAGGGUUUGAGCAG
MIMAT0003293	hsa-miR-624	hsa-miR-624*	UAGUACCAGUACCUUGUGUUCA
MIMAT0004807	N/A	hsa-miR-624	CACAAGGUAUUGGUAUUACCU
MIMAT0003298	hsa-miR-629	hsa-miR-629*	GUUCUCCCAACGUAAGCCCAGC
MIMAT0004810	N/A	hsa-miR-629	UGGGUUUACGUUGGGAGAACU

[1] The sequence of hsa-miR-340 is UCCGUCUCAGUUACUUUAUAGCC, whereas the sequence of hsa-miR-340* is UCCGUCUCAGUUACUUUAUAGC.

[2] The sequence of hsa-miR-422b is CUGGACUUGGAGUCAGAAGGCC, whereas the sequence of hsa-miR-378 is ACUGGACUUGGAGUCAGAAGG.
