# Supplementary material for: miRSystem: An Integrated System for Characterizing Enriched Functions and Pathways of MicroRNA Targets
Source: PLoS One. 2012 Aug 1;7(8):e42390. doi: 10.1371/journal.pone.0042390 (PMC3411648; doi:10.1371/journal.pone.0042390)
Supplement: Table S2 — Prediction algorithms and databases available in miRSystem (PDF) [file pone.0042390.s004.pdf]

Table S2 – Prediction algorithms and databases available in miRSystem

Source	Version[a]	Original Records	Mappable Number[b]
**DIANA-microT**	10	1,450,653	1,413,583
**miRanda**	11	927,867	910,940
**mirBridge[c]**	10	41,382	41,382
**PicTar[d]**	9.1	69,642	59,479
**PITA**	11	209,579	206,474
**rna22**	8	296,518	219,395
**TargetScan[e]**	16	110,284	110,284
**TarBase and miRecords[f]**	6-16	1,661	1,575

[a] The version mapped to miRBase.

[b] Number of mappable records in miRBase version 17.

[c] The records of miRBridge were collected from the supplementary data.

[d] The information about PicTar was obtained from UCSC Genome Browser.

[e] Families IDs were converted to single miRNA IDs to get complete miRNA-gene matching data.

[f] Merged together as experimentally validated datasets.
