# Supplementary material for: miRSystem: An Integrated System for Characterizing Enriched Functions and Pathways of MicroRNA Targets
Source: PLoS One. 2012 Aug 1;7(8):e42390. doi: 10.1371/journal.pone.0042390 (PMC3411648; doi:10.1371/journal.pone.0042390)
Supplement: Table S3 — The potential number of miRNA-gene pairs obtained with different combination of multiple algorithms (PDF) [file pone.0042390.s005.pdf]

Table S3 –The total potential number of miRNA-gene pairs obtained by tallying different prediction algorithms and experimentally validated data sources.

# of miRNA-gene pairs identified by 7 algorithms and 1 integrated validation database[a]	# of potential miRNA-gene pairs (%)	Cumulative percentage of miRNA-gene pairs
8	49 (0.002%)	0.002%
7	1,055 (0.050%)	0.052%
6	7,801 (0.366%)	0.418%
5	21,204 (0.996%)	1.415%
4	40,377 (1.897%)	3.311%
3	120,605 (5.666%)	8.978%
2	341,716 (16.054%)	25.031%
1	1,595,744 (74.969%)	100.000%
TOTAL	2,128,551 (100.00%)[b]	--

[a] two experimental validated data sources, TarBase and miRecords, were integrated into one database

[b] a total of 2,128,551 miRNA-gene pairs were included in miRSystem.
