# Supplementary material for: miRSystem: An Integrated System for Characterizing Enriched Functions and Pathways of MicroRNA Targets
Source: PLoS One. 2012 Aug 1;7(8):e42390. doi: 10.1371/journal.pone.0042390 (PMC3411648; doi:10.1371/journal.pone.0042390)
Supplement: Table S4 — Significantly expressed miRNAs (PDF) [file pone.0042390.s006.pdf]

Table S4 – Significantly expressed miRNAs

(A) GSE16558

miRNA name	*P*-value	Fold Change[a]
**hsa-miR-449**	$3.26*10^{-5}$	23.48
**hsa-miR-141**	$1.73*10^{-4}$	16.79
**hsa-miR-34a**	$5.08*10^{-4}$	12.86

[a]Fold change of expression in multiple myeloma cells versus normal control cells.

(B) GSE19536

miRNA name	*P*-value	Fold Change[a]
**hsa-miR-190b**	$1.58*10^{-11}$	3.64
**hsa-miR-342-3p**	$3.65*10^{-11}$	2.67
**hsa-miR-29c*[a]**	$1.43*10^{-10}$	2.35
**hsa-miR-149**	$6.55*10^{-10}$	3.66
**hsa-miR-29c**	$4.37*10^{-9}$	2.00
**hsa-miR-342-5p**	$4.56*10^{-9}$	2.60

[a]Fold change of expression in ER^+ versus ER^- cells.

[b]Excluded from further analysis because no gene targets were identified.
