# Supplementary material for: miRSystem: An Integrated System for Characterizing Enriched Functions and Pathways of MicroRNA Targets
Source: PLoS One. 2012 Aug 1;7(8):e42390. doi: 10.1371/journal.pone.0042390 (PMC3411648; doi:10.1371/journal.pone.0042390)
Supplement: Table S5 — Top 3 enriched pathways of the 3 miRNAs identified in GSE16558 by (A) functional annotation summary and (B) pathway ranking summary in miRSystem (PDF) [file pone.0042390.s007.pdf]

Table S5 –Top 3 enriched pathways of the 3 miRNAs identified in GSE16558 by (A) functional annotation summary and (B) pathway ranking summary in miRSystem

(A) Functional annotation summary

Database	Item	Gene	Raw[a]	Empirical[b]
**KEGG**	PATHWAY_IN_CANCER	26	$9.86*10^{-7}$	$5.69*10^{-4}$
**REACTOME**	PLATELET_ACTIVATION_SIGNALING_AND_AGGREGATION	17	$4.22*10^{-5}$	$6.76*10^{-4}$
**PID**	NOTCH_SIGNALING_PATHWAY	12	$5.07*10^{-6}$	$7.28*10^{-4}$

[a] Raw *P*-values were obtained by a hypergeometric test.

[b] Empirical *P*-values were compared with 1,000 random selections.

(B) Pathway ranking summary

Database	Item	Gene	Score[a]
**REACTOME**	AXON_GUIDANCE	61	9.108
**REACTOME**	DEVELOPMENTAL_BIOLOGY	81	7.156
**REACTOME**	L1CAM_INTERACTIONS	27	6.500

[a] Score was calculated based on the weighted pathway-ranking method described in "Methods".
