# Supplementary material for: miRSystem: An Integrated System for Characterizing Enriched Functions and Pathways of MicroRNA Targets
Source: PLoS One. 2012 Aug 1;7(8):e42390. doi: 10.1371/journal.pone.0042390 (PMC3411648; doi:10.1371/journal.pone.0042390)
Supplement: Table S6 — Top 3 enriched pathways of the 5 miRNAs identified in GSE19536 by (A) functional annotation summary and (B) pathway ranking summary in miRSystem. (PDF) [file pone.0042390.s008.pdf]

Table S6 –Top 3 enriched pathways of the 5 miRNAs identified in GSE19536 by (A) functional annotation summary and (B) pathway ranking summary in miRSystem

(A) Functional annotation summary

Database	Item	Gene	Raw[a]	Empirical[b]
**REACTOME**	APOPTOSIS	8	$1.33*10^{-5}$	$7.59*10^{-4}$
**REACTOME**	AXON_GUIDANCE	11	$4.23*10^{-6}$	$1.74*10^{-3}$
**REACTOME**	DEVELOPMENTAL_BIOLOGY	13	$6.35*10^{-5}$	$4.84*10^{-3}$

[a] Raw *P*-values were obtained by a hypergeometric test.

[b] Empirical *P*-values were compared with 1,000 random selections.

(B) Pathway ranking summary

Database	Item	Gene	Score[a]
**REACTOME**	DEVELOPMENTAL_BIOLOGY	91	4.035
**REACTOME**	AXON_GUIDANCE	61	3.732
**KEGG**	FOCAL_ADHESION	47	3.044

[a] Score was calculated based on the weighted pathway-ranking method described in "Methods".
